# Feature selection environment for genomic applications

**DOI:** 10.1186/1471-2105-9-451

**Published:** 2008-10-22

**Authors:** Fabrício Martins Lopes, David Corrêa Martins, Roberto M Cesar

**Affiliations:** 1 Instituto de Matemática e Estatística, Universidade de São Paulo, Rua do Matão 1010, 05508-090, São Paulo-SP, Brazil; 2 COINF, Universidade Tecnológica Federal do Paraná, Av. Alberto Carazzai, 1640, 86300-000, Cornélio Procópio-PR, Brazil

## Abstract

**Background:**

Feature selection is a pattern recognition approach to choose important variables according to some criteria in order to distinguish or explain certain phenomena (i.e., for dimensionality reduction). There are many genomic and proteomic applications that rely on feature selection to answer questions such as selecting signature genes which are informative about some biological state, e.g., normal tissues and several types of cancer; or inferring a prediction network among elements such as genes, proteins and external stimuli. In these applications, a recurrent problem is the lack of samples to perform an adequate estimate of the joint probabilities between element states. A myriad of feature selection algorithms and criterion functions have been proposed, although it is difficult to point the best solution for each application.

**Results:**

The intent of this work is to provide an open-source multiplataform graphical environment for bioinformatics problems, which supports many feature selection algorithms, criterion functions and graphic visualization tools such as scatterplots, parallel coordinates and graphs. A feature selection approach for growing genetic networks from seed genes (targets or predictors) is also implemented in the system.

**Conclusion:**

The proposed feature selection environment allows data analysis using several algorithms, criterion functions and graphic visualization tools. Our experiments have shown the software effectiveness in two distinct types of biological problems. Besides, the environment can be used in different pattern recognition applications, although the main concern regards bioinformatics tasks.

## Background

Pattern recognition methods allow the classification of objects or patterns in a number of classes [1,2]. In statistical pattern recognition, given a set of classes and an unknown object, a pattern recognition system associates the object to one of the classes, based on defined measures in a feature space. In many applications, specifically in bioinformatics, the feature space dimension tends to be very large, making difficult both classification tasks and network inference. In order to overcome this inconvenient situation, the study of dimensionality reduction problem in pattern recognition is very important.

The so called "curse of dimensionality" [3] is a phenomenon in which the number of training samples required to a satisfactory classification performance or network inference is given by an exponential function of the feature space dimension. This is the main motivation by which performing dimensionality reduction is important in problems with large number of features and small number of training samples. Many applications in bioinformatics belong to this context. Data sets containing mRNA transcription expressions from microarray or SAGE, for example, present thousands of genes (features) and only some dozens of samples that may come from cell states or types of tissues.

In this context, this work presents an open-source feature selection environment, which includes different search algorithms and criterion functions. Due to the curse of dimensionality phenomenon, error estimation is a crucial issue. We have developed two ways to embed error estimation in the criterion functions, one based on classification error and another based on classification information. The main idea is based on the penalization of non-observed or rarely observed instances. After the feature selection, it is possible to apply classical error estimation methods like resubstitution and holdout cross-validation. Illustrative results obtained on gene regulation networks inference and on classification of breast cancer cells using the proposed software are included in the present paper.

## Methods

### Data preprocessing

A critical issue in gene expression data analysis is the pre-processing of the data. Depending on the criterion function used, a step of data quantization may be required if the original input data is not discrete. The quantization usually requires a normalization of the data. In the software, the *normal transformation *is implemented. It consists in subtracting the signal (expression profile) by its mean and then dividing by its standard deviation. Formally, for every signal *g*(*t*), its equation is given by $\eta [g(t)]=\frac{g(t)-E[g(t)]}{\sigma [g(t)]}$, where *E*[*g*(*t*)] and *σ*[*g*(*t*)] are, respectively, the expectation and standard deviation of *g*(*t*). There are two options: 1 – normalization by feature (*g *is a feature and *t *is a sample); 2 – normalization by sample (*g *is a sample and *t *is a feature).

The quantization of a normalized signal *η*[*g*(*t*)] (*g *being feature or sample depending on the chosen option for normalization) is a mapping from the continuous expression into *k *qualitative expressions {0, 1, ..., *k *- 1}. It is performed by a threshold mapping that divides the interval given by the minimum and the maximum values of *g *in *k *intervals of equal size. A given continuous value *r *of *η*[*g*(*t*)] is mapped to an integer *i *- 1, where *i *is the *i*-th interval to which *r *belongs.

### Implemented feature selection algorithms

The simplest implemented feature selection algorithm is the exhaustive search. This algorithm searches the whole search space. As a result, the selected features are optimal according to the criterion function chosen to guide the algorithm. However, the computational cost often makes this approach inadequate, thus requiring alternative (non-optimal) algorithms.

In this work we have implemented two sub-optimal approaches with unique solution, being also known as wrappers [4]. In the first one, the selected subset starts empty and features are inserted by optimizing a criterion function until a stop condition is satisfied, which is often based on the subset size or a threshold. This method is known as Sequential Forward Selection (SFS) [5]. Although simple and efficient, SFS presents an undesirable drawback known as nesting effect. This effect arises because the selected features are never discarded.

**Figure 1:**
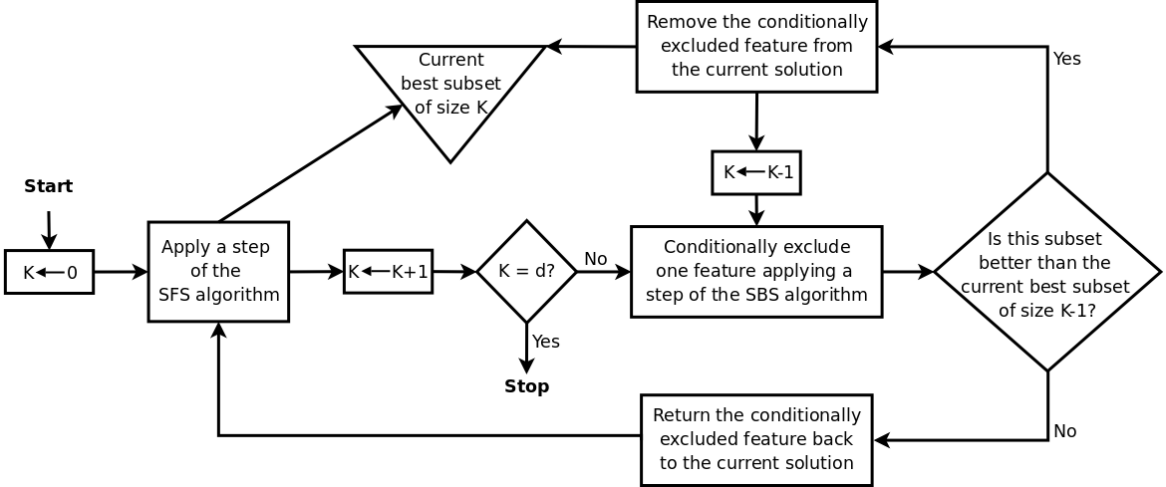
**Flowchart of the SFFS algorithm**. Simplified flowchart of the SFFS algorithm (adapted from [21]).

In order to circumvent the nesting effect, the Sequential Floating Forward Selection (SFFS) is also available in the software. The SFFS algorithm may be formalized as in [5]. A schematic flowchart of the SFFS algorithm is presented in Figure 1. In this algorithm, the SFS and the SBS (Sequential Backward Search – a counterpart of the SFS) are successively applied.

Considering the adopted feature selection and criterion function, the intermediate results are stored in a list. The best sets of this list are displayed as the algorithm result.

Other feature selection methods will be implemented, both wrappers (e.g., "plus l-take away r", simulated annealing, genetic algorithm) and filters (e.g., correlation-based, consistency-based, entropy-based), and embedded methods (e.g., CART) [3,4,6,7]. Also, methods for feature extraction, such as PCA and ICA [8,9], will be implemented. We hope that the availability of the software as open-source will help the inclusion of other functions by the international community.

### Implemented criterion functions

We implemented criterion functions based on classification information (mean conditional entropy) and classification error (Coefficient of Determination [10]), introducing some penalization on poorly or non-observed patterns. Introducing penalization on rarely observed instances embeds the error estimation into the criterion functions. This approach has two main advantages. First, the selected features are obtained not only by their informative content about the classes, but also by the number of well observed instances they have (large number of well observed instances means small error estimation). Another advantage is that the right dimension of the feature subset is also estimated (the dimension parameter is not required). In the software, the implemented feature selection algorithms (SFS and SFFS) requires the dimension parameter just to limit the execution time, performing the procedures until the subset size reaches that parameter. According to the criterion function values, the resulting subsets can have smaller dimension than the specified by the parameter.

#### Mean conditional entropy

Information Theory, originated by Shannon's works [11], can be employed on feature selection problems [2]. The mean conditional entropy of *Y *given all the possible instances **x **∈ **X **is given by:

(1) $$H(Y|X)=\sum_{x\in X}P(x)\;H(Y|x),$$

where *P*(*Y *| **x**) is the conditional probability of *Y *given the observation of the instance **x**, *H*(*Y *| **x**) = -*Σ*_*y*∈*Y *_*P*(*y *| **x**) log *P*(*y *| **x**). Shannon's entropy can be generalized by using Tsallis Entropy [12] with a parameter *q*. We have that *q *= 1 is the Shannon's Entropy (extensive entropy), *q *< 1 is the sub-extensive entropy and *q *> 1 is the super-extensive entropy. The Tsallis Entropy is given by $H(Y|x)=\frac{1}{q-1}(1-\sum_{y\in Y}{\left(P(Y|x)\right)}^{q})$ Lower values of *H *yield better feature subspaces (the lower *H*, the larger is the information gained about *Y *by observing **X**).

#### Coefficient of Determination

The Coefficient of Determination (CoD) [10], like the conditional entropy, is a non-linear criterion useful for feature selection problems [13]. The CoD is given by:

(2) $${\text{CoD}}_{Y}(X)=\frac{\varepsilon_{Y}-\varepsilon_{Y}(X)}{\varepsilon_{Y}},$$

where *ε*_*Y *_= 1 - max_*y*∈*Y *_*P*(*y*) is the prior error, i.e., the error by predicting *Y *in the absence of other observations, and *ε*_*Y*_(**X**) = Σ_**x∈X **_*P*(**x**) (1 - max_*y*∈*Y *_*P*(*y *| **x**)) is the average error by predicting *Y *based on the observation of **X**. Larger values of CoD yield to better feature subspaces (CoD = 0 means that the feature subspace does not improve the prior error and CoD = 1 means that the error is fully eliminated).

#### Penalization of non-observed instances

In order to embed the error estimation, due to feature vectors with large dimensions and insufficient number of samples, we adopted a strategy to involve non-observed instances in the criterion value calculus [14]. A positive probability mass is attributed to the non-observed instances and the corresponding contribution is the same as observing only the *Y *values with no other observations. In the case of mean conditional entropy, the non-observed instances receive the entropy equal to *H*(*Y*) whereas the CoD receives the prior error *ε*_*Y *_value. The probability mass for the non-observed instances is parameterized by *α*. This parameter is added to the absolute frequency (number of occurrences) of all possible instances. So, the mean conditional entropy with this type of penalization becomes:

(3) $$\begin{array}{c} H(Y|X)=\frac{\alpha (M-N)H(Y)}{\alpha M+s}+\\ \frac{\sum_{i=1}^{N}\left(f_{i}+\alpha )H(Y|X=x_{i}\right)}{\alpha M+s}, \end{array}$$

where *M *is the number of possible instances of the feature vector **X**, *N *is the number of observed instances (so, the number of non-observed instances is given by *M *- *N*), *f*_*i *_is the absolute frequency (number of observations) of **x**_**i **_and *s *is the number of samples.

The CoD becomes:

(4) $$\begin{array}{c} {\text{CoD}}_{Y}(X)=1-\frac{\alpha (M-N)}{\alpha M+s}-\\ \frac{\sum_{i=1}^{N}\left(f_{i}+\alpha )(1-\max_{y\in Y}P(y|x_{i})\right)}{(\alpha M+s)\varepsilon_{Y}}. \end{array}$$

#### Penalization of rarely observed instances

In this penalization, the non-observed instances are not taken into account. This penalization consists in changing the conditional probability distribution of the instances that have just a unique observation [15]. It makes sense because if an instance **x **has only 1 observation, the value of *Y *is fully determined (*H*(*Y *| **X **= **x**) = 0 and CoD_*Y*_(**X**) = 1), but the confidence about the real distribution of *P*(*Y *| **X **= **x**) is very low. A parameter *β *gives a confidence value that *Y *= *y*. The main idea is to equally distribute 1 - *β *over all *P*(*Y *≠ *y *| **X **= **x**) and to attribute *β *to *P*(*Y *= *y *| **X **= **x**). In Barrera *et al *[15], the *β *value is $\frac{1}{\left|Y\right|}$ where |*Y*| is the number of classes (cardinality of *Y*), i.e., the uniform distribution (strongest penalization). By adapting this penalization to the Equation (1), the mean conditional entropy becomes:

(5) $$\begin{array}{c} H(Y|x)=\frac{M-N}{s}H((F(0),...,F(|Y|-1)))+\\ \sum_{x\in X:P(x)>\frac{1}{s}}P(x)\;H(Y|x), \end{array}$$

where *F *: {0, 1, ..., |*Y*| - 1} → [0, 1] is the probability distribution given by

$$F(i)=\{\begin{array}{cc} \beta & \text{for }i=y,\\ \frac{1-\beta }{|Y|-1} & \text{for }i\neq y, \end{array}$$

and *N *is the number of instances **x **with $P(x)>\frac{1}{s}$ (more than one observation).

Since *ε*_*Y*_(**x**) = 1 - *β *when $P(Y|x)=\frac{1}{s}$, the CoD with this penalization is given by:

(6) $$\begin{array}{c} {\text{CoD}}_{Y}(X)=1-\frac{\left(M-N)(1-\beta \right)}{s\varepsilon_{Y}}-\\ \frac{\sum_{x\in X:P(x)>\frac{1}{s}}P(x)(1-max_{y\in Y}P(y|x))}{\varepsilon_{Y}}. \end{array}$$

### Classifier design and generalization

After the feature selection, the classifier is designed from the table of conditional probabilities of the classes (*Y*) given the patterns (**X**). This table is used as a Bayesian classifier where, for each given instance, the chosen label is the one with maximum conditional probability for the considered instance. In case of ties, i.e., instances that have two or more labels of maximum probability (including non-observed instances), generalization according to some criterion is carried out. A commonly used criterion is the nearest neighbors with some distance metric [1,2]. The nearest neighbors using Euclidean distance has been implemented for generalization. In this implementation, the nearest neighbors with equal distances are taken and the occurrences of each label given these patterns are summed. If the tie persists, the next nearest neighbors are taken and the process is repeated until only one of such labels has the maximum number of occurrences (i.e., until the tie is broken). The label with the maximum number of occurrences at the end of this process is chosen as the class to which the considered instance belongs.

There are many classifiers described in the literature, such as Parzen, Fisher Linear Discriminant, Binary Decision Tree, Perceptron, Support Vector Machines and so on [3]. The software has been designed so that such additional classifiers may be incorporated. One of the objectives of the software is to implement as many classifiers as possible. However, the primary focus is given on feature selection algorithms and criterion functions.

### Error estimation methods

In order to validate and compare feature selection algorithms, error estimation methods are required, especially for data with high dimensionality and small number of samples which is usually the case in bioinformatics problems.

The first method implemented in the software is the simple resubstitution in which the training set is also used as testing set. For many bioinformatics problems, the resubstitution error estimation works fine since usually there is not enough data samples to perform a good estimation, and partitioning the data leads to an even smaller training sample size (see [16] for a detailed discussion).

The Holdout cross validation is also available in the software. It consists in partitioning the data in two sets: training and test sets. The size of the training set can be defined in terms of percentage of total data samples. Thus, the feature selection method is applied to the training set, the classifier is designed taking into account the resulting feature set and the conditional probability distributions given by the training set. Finally, the designed classifier is applied to the test set and the classification error rate is obtained. Alternative error estimation methods like K-fold cross-validation, leave-one-out (a particular kind of cross-validation) and bootstrap will be implemented in the software.

### Network inference

If one considers the training set as time series data, i.e., each sample representing a state of the system at time *t*, it is possible to generate networks of prediction based on the Probabilistic Genetic Network model described in [15]. This model is a Markov chain that mimics the properties of a gene as a non-linear stochastic gate. Given a fixed gene *Y *as target, the general idea of this model is to determine the subset of genes **Z **⊆ **X **that makes the best prediction of the *Y *value in the next instant of time. In order to achieve this intent, the system is considered translation invariant, i.e., the transition function is the same for every time instant *t*. In this way, it is possible to apply a feature selection method for every target, since a sample is represented by the pair (**X**[*t*], *Y*[*t *+ 1]) and the training set is composed by a collection of these samples along the time domain (so, the number of samples is given by *t *- 1). After the application of the feature selection process for every target, the best feature subsets obtained for each target gene define the network connections.

### Software description

**Figure 2:**
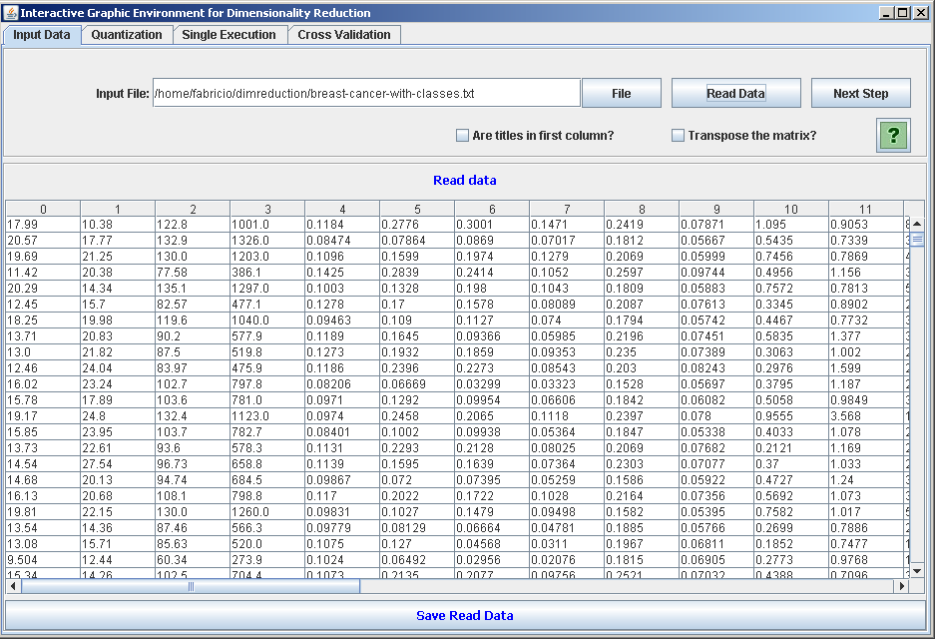
**Upload biological data**. The software panel to upload biological data.

**Figure 3:**
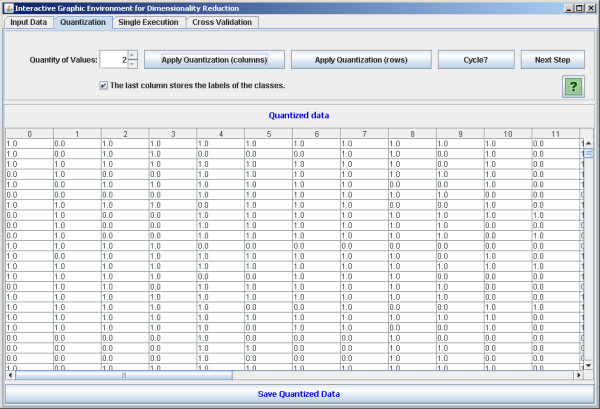
**Quantization process**. The software panel to apply quantization process.

**Figure 4:**
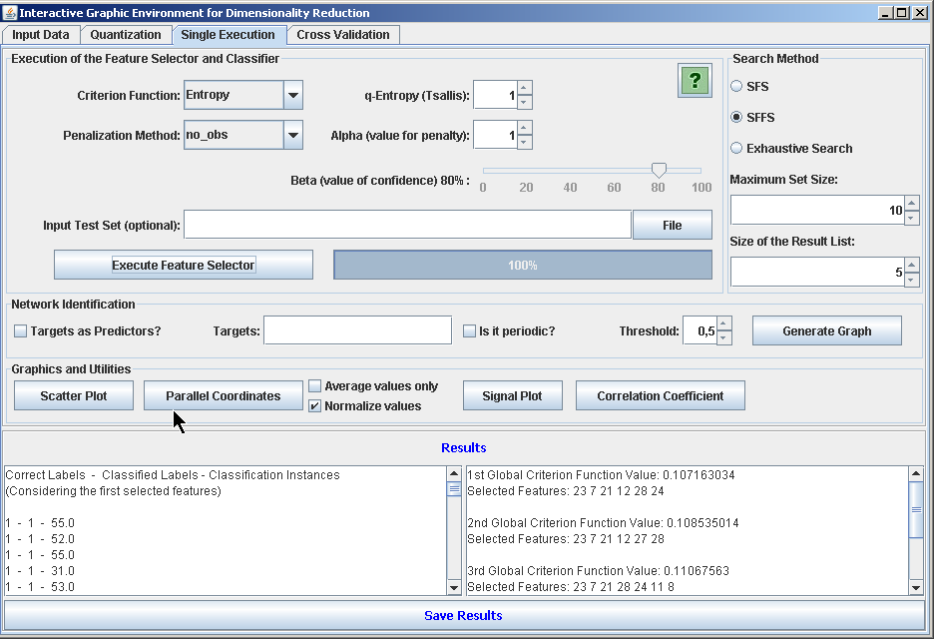
**Single feature selection execution**. The software panel to apply single feature selection execution and feature selection approach for growing of genetic networks from target genes.

The software is implemented in Java in order to be executable in different platforms. It is open source and intended to be continuously developed in a world-wide collaboration. There are four main panels: the first one allows the user to load the data set (Figure 2). The second is optional for the user to define a quantization degree to the data set. The quantized data may be visualized (Figure 3). It is worth noting that the mean conditional entropy and the CoD require data quantization to discrete values. This fact explains the quantization step available in the software. Please refer to the Section *Data Preprocessing *for further information on the normalization and quantization methods applied. The reason to keep the quantization optional is because other criterion functions, such as the distance-based criteria, do not require quantization and the software is intended to implement as many criterion functions as possible. The third panel is dedicated to perform single tests (Figure 4). With this panel, the user can execute feature selection methods (see Section *Implemented feature selection algorithms*) along with the criterion function (see Section *Implemented criterion functions*). The user has two options: the first one is to apply the simple resubstitution method (i.e., to use the same input data as training and test set); the second option consists in to apply the classifier on a different data set given by the user specified at input test set text box (Holdout cross-validation with training and test sets partitioning defined by the user).

**Figure 5:**
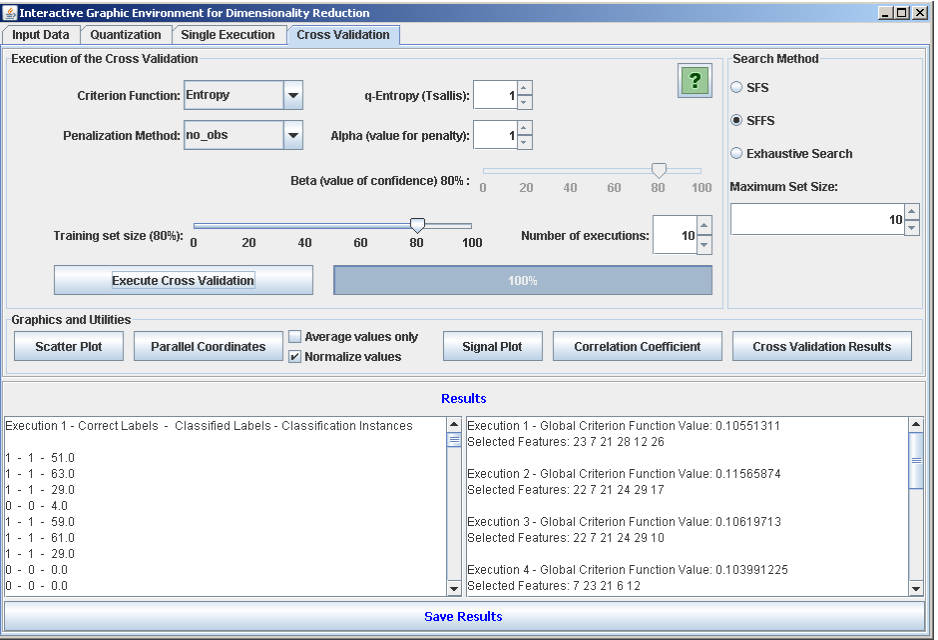
**Cross-validation**. The software panel to apply feature selection execution using cross-validation.

The fourth panel is very similar to the previous one, but allows the application of automatic cross-validation procedures [17] (Figure 5) (see Section *Error estimation methods*). This panel presents only the Holdout cross-validation with training and test sets partitioning performed randomly.

There are other implemented utilities, including the visualization of the results of the feature selection as scatterplots, parallel coordinates [18] and graphs. Every panel has its own help button that activates a popup window explaining the functionalities and options regarding the corresponding panel. Also, description boxes are displayed when the mouse cursor is placed over some option in order to quickly recall the user about the functionalities of the system.

## Results and discussion

This section presents some illustrative results in two different typical situations in bioinformatics. All results shown here were obtained by using the default values in the software: SFFS with mean conditional entropy and penalization of non-observed instances, *α *= 1, Tsallis parameter *q *= 1, and maximum set size = 10.

**Figure 6:**
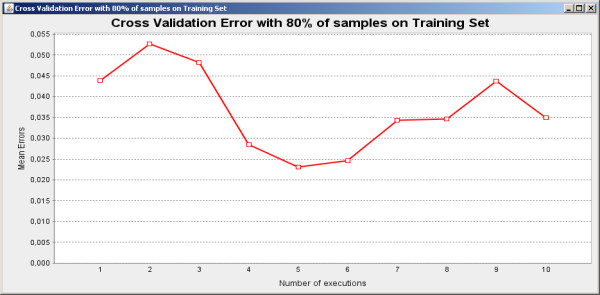
**Cross-validation results**. Illustrative cross-validation results using 10 executions, 80% of data as training set and 20% as test set.

**Figure 7:**
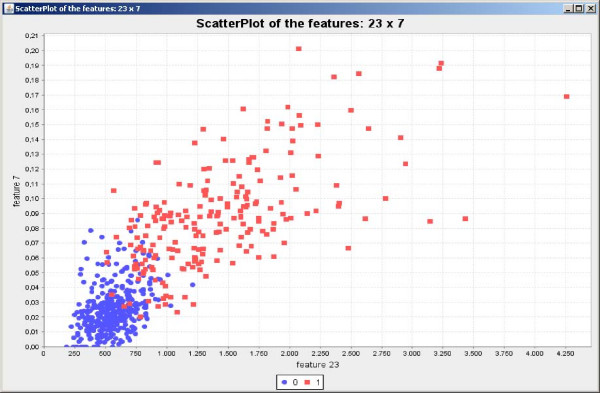
**Scatterplot generated over the benign and malignant tissues data set**. Scatterplot generated by using the first two features included in the SFFS result over the benign and malignant tissues data set.

**Figure 8:**
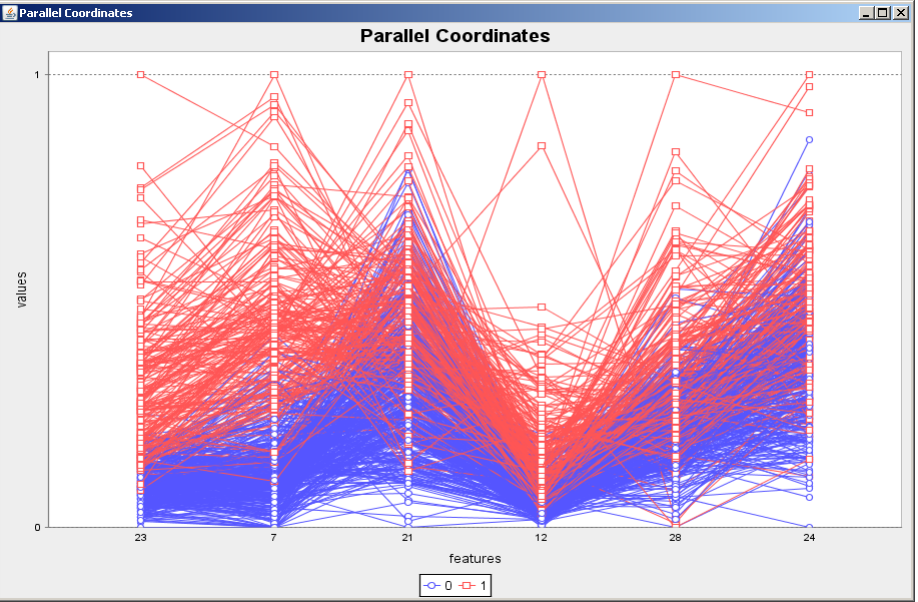
**Parallel coordinates generated over the benign and malignant tissues data set**. Parallel coordinates generated by using the six features included in the SFFS result over the benign and malignant data set. The original values were normalized between 0 and 1.

Initially the software was applied for feature selection in a biological classification problem to classify breast cancer cells [19] in two possible classes: benign and malignant. The training set is composed by 589 instances and 32 features. The SFFS algorithm with the entropy criterion was explored. The results shown in Figure 6 present very low variations and high accurate classification achieving 96.30% of accuracy on average. The scatterplot taking the first two features of the feature set result obtained by SFFS is shown in Figure 7, while the parallel coordinates of the resulting six features are shown in Figure 8.

**Figure 9:**
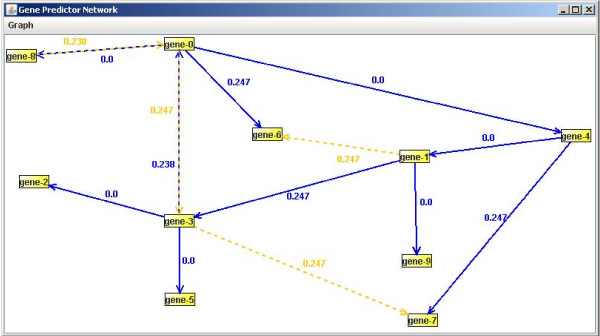
**Results of the growing genetic networks from target genes**. Identified network: dashed lines represent the false positives and solid lines the positives. There are no false negatives.

The second addressed computational biology problem is genetic network identification. In this case we used an artificial genetic network generated by the approach presented in [20]. The adopted parameters were: 10 nodes, binary quantization, 20 transitions (instants of time), 1 average degree per node and random graphs of Erdös-Rényi. Figure 9 presents the network identification, in which no false negatives occurred and just few false positives. The methodology of network generation is described in Section *Network inference*, the same as used to generate probabilistic genetic networks of the *Plasmodium falciparum *from microarray data [15], thus showing the possibility of exploring the software in such an important problem in bioinformatics.

## Conclusion

The proposed feature selection environment allows data analysis using several algorithms, criterion functions and graphic visualization tools. Our experiments have shown the software effectiveness in two distinct types of biological problems. Besides, the environment can be used in different pattern recognition applications, although the main concern regards bioinformatics tasks, especially those involving high-dimensional data (large number of genes, for example) with small number of samples. Users without programming skills are allowed to manipulate the software in an easy way, just by clicking to select file inputs, quantization, algorithms, criterion functions, error estimation methods and visualization of the results, while an intuitive help system quickly presents the instructions of the present functionalities that the user may look for. On the other hand, the availability of the software as open-source allows programmers to explore the implemented methods as libraries in their own programs. The environment is implemented in "wizard style", i.e., with tabs delimiting each procedure. A complete technical report that complements the help information is available at the project web site. Two video demos of the software in use are available at .

This software opens space for future work. The next steps consist in the implementation of other classical feature selection algorithms, including wrappers, filters and embedded methods, criterion functions (e.g., based on distances between classes [1]); classifier design methods like Parzen, Fisher Linear Discriminant, Binary Decision Tree, Perceptron and Support Vector Machines; error estimation methods such as leave-one-out, K-fold cross-validation and bootstrap; and the inclusion of classical methods of feature extraction, such as PCA and ICA [8,9].

## Availability and requirements

• Project name: DimReduction

• Project home pages:  and 

• Operating system: Platform independent

• Programming language: Java

• Other requirements: Java Runtime Environment (JRE) 1.5 or higher

• License: code available; GNU Lesser General Public License

• Video demos: 

• Complete technical report: 

## Authors' contributions

FML and DCM wrote the software code, analyzed the data and wrote the manuscript. RMCJ participated in the design and coordination of the study. All authors contributed to, read and approved the final manuscript.
