# Feature selection environment for genomic applications

**DOI:** 10.1186/1471-2105-10-285

**Published:** 2009-09-10

**Authors:** Fabrício Martins Lopes, David Corrêa Martins, Roberto M Cesar

**Affiliations:** 1 Instituto de Matemática e Estatística, Universidade de São Paulo, Rua do Matão 1010, 05508-090, São Paulo-SP, Brazil; 2 COINF, Universidade Tecnológica Federal do Paraná, Av. Alberto Carazzai, 1640, 86300-000, Cornélio Procópio-PR, Brazil

## Correction

We have found an error after the publication of this work [1]. The error is present in the following sentence: "We have that *q *= 1 is the Shannon's Entropy (extensive entropy), *q *< 1 is the sub-extensive entropy and *q *> 1 is the super-extensive entropy".

Where it says "sub-extensive", should be changed to "super-extensive" and where it says "super-extensive", should be changed to "sub-extensive".

The corrected sentence: We have that *q *= 1 is the Shannon's Entropy (extensive entropy), *q *< 1 is the super-extensive entropy and *q *> 1 is the sub-extensive entropy.

We regret any inconvenience that this inaccuracy in the text might have caused.

## References

[B1] Lopes FM, Martins DC, Cesar RM (2008). Feature selection environment for genomic applications. BMC Bioinformatics.

